# The relevance of phenotypic definition in treatment resistant forms of major depressive disorder: a narrative review

**DOI:** 10.3389/fphar.2026.1819237

**Published:** 2026-06-12

**Authors:** Pasquale Paribello, Ulker Isayeva, Silvia Lazzardi, Marco Pinna, Martina Contu, Anna Meloni, Claudia Pisanu, Alessio Squassina, Bernhard T. Baune, Mirko Manchia

**Affiliations:** 1 Section of Psychiatry, Department of Medical Sciences and Public Health, University of Cagliari, Cagliari, Italy; 2 Unit of Clinical Psychiatry, Department of Specialistic Medicine, University Hospital Agency of Cagliari, Cagliari, Italy; 3 Section of Neuroscience and Clinical Pharmacology, Department of Biomedical Sciences, University of Cagliari, Cagliari, Italy; 4 Department of Psychiatry, University Hospital Muenster, Münster, Germany; 5 Department of Psychiatry, University of Melbourne, Melbourne, VIC, Australia; 6 The Florey Institute of Neuroscience and Mental Health, Melbourne, VIC, Australia; 7 Department of Pharmacology, Dalhousie University, Halifax, NS, Canada

**Keywords:** biomarkers, major depressive disorder, measurement-based care, precision psychiatry, treatment-resistant depression

## Abstract

Treatment-resistant depression (TRD) represents a major clinical and public health challenge contributing disproportionately to disability, healthcare utilisation, and societal costs. Progress in the field may be hindered by substantial heterogeneity in TRD phenotypic definitions and inconsistencies in their operationalisation across research and clinical settings. In this narrative review, we provide an updated overview of TRD research, focusing on limitations of current phenotypic frameworks, key sources of heterogeneity, and emerging biologically informed predictive approaches. We examine how variability in clinical assessment, comorbidities, treatment adherence, and adequacy of therapeutic trials may contribute to pseudo-resistance and consequent phenotypic instability, given that current TRD definitions largely rely on the number of failed antidepressant trials. Evidence on genetic, inflammatory, cognitive, and personality correlates of TRD is also summarised, highlighting both promising signals and persisting gaps. In addition, we discuss the potential role of measurement-based care and algorithm-guided treatment strategies in improving TRD identification and management. Overall, convergence toward more standardised TRD phenotyping—particularly through systematic assessment of adherence and symptom dimensions—appears essential to enhance ecological validity, support precision psychiatry, and advance translational research.

## 1 Introduction

Major depressive disorder (MDD) is a complex, heterogeneous and common disorder affecting approximately 280 million people worldwide (Author anonymous, 2025; Marx et al., 2023). MDD represents a leading cause of disability globally (Author anonymous, 2018), as shown by the increase in the number of all-ages years lost to disability (YLD) attributed to MDD alone, estimated at 32% from 1990 to 2007 and by an additional 12.6% from 2007 to 2017. Data from the US suggest a growth in MDD prevalence over time (Goodwin et al., 2022), which is unevenly distributed by age, with the greatest increase experienced among individuals between 18 and 34 years of age (Proudman et al., 2021). Similarly, the economic burden associated with MDD has also raised, but only 40% of the overall increase in cost appears to be merely attributable to the increase in prevalence, whilst the remaining appears to be associated with an increased cost per person (e.g., absenteeism, presenteeism cost in the workplace) (Greenberg et al., 2021). Within this spectrum, treatment-resistant depression (TRD) constitutes a particularly severe manifestation, characterised by poorer clinical outcomes, greater functional impairment, and markedly higher healthcare utilisation and costs compared to treatment-responsive forms of MDD. TRD is a not infrequent manifestation of MDD, representing a major challenge in mental health, affecting a significant number of patients, and aggravating the clinical and socio-economic burden of MDD. Indeed, the manifestation of TRD significantly impacts on the MDD burden estimates, with substantial increase in medical expenditures (Olchanski et al., 2013). TRD is associated with a 29.3% higher medical costs compared with patients with treatment responsive forms of MDD (Olchanski et al., 2013), and data from diverse patient populations such as Veterans (Szukis et al., 2021) or from outpatient settings (McCrone et al., 2018) converge in showing that these costs are related also to a more intensive use of healthcare resources and to lower levels of general functioning. Interestingly, these findings were recently confirmed by a large EU-based survey which showed that TRD patients had significantly lower quality of life, greater impairment in work productivity and activity, and increased healthcare resource utilisation as compared with non-TRD patients and the general population (Jaffe et al., 2019). Thus, MDD and its more severe clinical form, TRD, exert an enormous clinical and socio-economic toll that prompts research attempts to identify at-risk individuals early in the course of the illness, modifying determinants and formulating preventative interventions. Despite its clinical relevance, progress in understanding and managing TRD has been hindered by substantial heterogeneity in its phenotypic definition and inconsistencies in its operationalisation across clinical and research settings. Current definitions largely rely on the number of failed antidepressant trials, often without adequately accounting for treatment adherence, adequacy of therapeutic trials or systematic assessment of symptom change. This introduces significant phenotypic instability, including the risk of misclassifying pseudo-resistance as true treatment resistance, and limits both clinical decision-making and the comparability of research findings.

The etiological factors contributing to TRD are complex, multifactorial and not yet fully understood (McIntyre et al., 2023). Attention has been paid to the genetic architecture of TRD with recent advances in the identification of genetic predictors (Koch et al., 2025; Xiong et al., 2025). Importantly, the clinical and neurobiological complexity of TRD could be grasped by adequately powered multimodal predictive models applying artificial intelligence analytical tools, such as machine learning, to identify individuals at higher or lower likelihood of responding to antidepressant treatments (Baune et al., 2024). These attempts are producing encouraging results, identifying a neuroimmune subtype of TRD (Sirés et al., 2025) potentially actionable with specific therapeutics. However, the clinical utility of these findings remains constrained, in part because biological signals are mapped onto heterogeneous and inconsistently defined phenotypes, reducing their reproducibility and translational applicability. Addressing this gap requires a shift toward more standardised and operationalised approaches to TRD definition and assessment. Strategies such as measurement-based care (MBC) and algorithm-guided treatment offer the potential to improve the consistency of symptom measurement, enhance detection of non-responsiveness, and reduce clinical inertia. While these approaches do not directly resolve the underlying biological heterogeneity of TRD, they may represent a critical step toward improving phenotypic precision, thereby enabling both more effective clinical management and more robust translational research. In this context, phenotypic instability emerges as a central barrier linking clinical uncertainty and limited translational progress.

## 2 Aims

This narrative review aims to examine the extent to which heterogeneity in the phenotypic definition and operationalisation of TRD limits progress in both clinical practice and translational research. Specifically, we seek to analyse sources of variability in current TRD definitions, including diagnostic heterogeneity, treatment adequacy and adherence (Marx et al., 2023); evaluate how these limitations impact the identification and interpretation of biological correlates of TRD, and discuss the potential role of structured approaches such as MBC and algorithm-guided treatment in improving phenotypic precision (GBD 2017 Disease and Injury Incidence and Prevalence Collaborators, 2018). By integrating clinical and biological perspectives, this review aims to strengthen the concept that improving the validity and consistency of TRD phenotyping represents a necessary step for advancing both treatment optimisation and biologically informed prediction models, rather than treating these domains as independent lines of investigation. To this end, we will first discuss the role of clinical heterogeneity in the definition of an accurate TRD phenotype, present the limitations of the current phenotypic definition of TRD, describe the neurobiological and genetic correlates of TRD, and give an overview of some possible future directions for the field. We argue that improving the validity and operationalisation of TRD phenotyping is a prerequisite for both effective clinical management and meaningful biological discovery.

## 3 Methods

For this narrative review, a targeted, unsystematic search of the literature was conducted on PubMed/MEDLINE, Scopus. The search included studies published up to January 2026 without formal start date restrictions. Search terms included combinations of “treatment-resistant depression”, “major depressive disorder”, “measurement-based care”, “clinical inertia”, “treatment algorithm”, “cognitive dysfunction”, “personality traits”, “treatment adherence”, “therapeutic drug monitoring”. Priority was given randomised controlled trials, and large observational studies. Relevant meta-analyses and systematic reviews were evaluated to search for additional sources significant for our purposes. Additional relevant articles were identified through manual reference screening of selected papers. Given the narrative nature of the review, formal inclusion/exclusion criteria were not predefined; however, article selection was guided by relevance to the core themes and prioritisation of high-quality evidence (RCTs, meta-analyses, large observational studies).

## 4 Phenotypic definition of treatment resistance in MDD

### 4.1 The clinical heterogeneity of MDD

The definition of TRD is inherently dependent on the underlying definition of MDD, which itself is characterised by substantial heterogeneity (Nunes et al., 2020). As a result, TRD represents a second-order construct built on an already unstable clinical phenotype, in which limitations in MDD definition are compounded by additional constraints imposed by treatment-based criteria. This creates a “double-layer” problem of phenotypic instability, in which variability in symptom expression, diagnostic practices, treatment adequacy, and adherence collectively undermines the reliability and validity of the TRD construct. For clarity, sources of heterogeneity can be broadly grouped into four domains: (i) nosological and diagnostic heterogeneity, (ii) symptom-level and biological heterogeneity, (iii) clinical and comorbidity-related heterogeneity, and (iv) longitudinal heterogeneity.

#### 4.1.1 Nosological and diagnostic heterogeneity

At the diagnostic level, MDD encompasses a highly heterogeneous set of symptom constellations. The nosological classification of MDD symptoms and its accuracy and reliability have been inherently hindered by this clinical heterogeneity. This nosological variability implies that individuals sharing the same diagnosis may differ substantially in symptom profiles, underlying biology and treatment response. According to the MDD criteria for the Diagnostic and Statistical Manual of Mental Disorders-5 edition (DSM-5), hundreds of different possible combinations are possible (Buch and Liston, 2021), with similar figures even when considering other possible categorical sets of criteria currently in use. Therefore, even if specific symptom constellations were associated with distinct biological substrates, substantial heterogeneity would persist within diagnostically defined groups. An additional source of heterogeneity may depend on the inter-rater reliability of diagnosis, which is substantially low for MDD. In fact, the field studies for the DSM-5 tested the diagnostic reliability for 27 different conditions, resulting in the identification of five entities with questionable or unacceptable reliability levels according to the current criteria (Vanheule et al., 2014). MDD presented a κ Cohen = 0.28, corresponding to a questionable reliability level. These sobering results are especially significant when considering the progressive relaxation over time for the reliability criteria thresholds (as a reference for comparison, Spitzer and Fleiss in 1974 proposed to consider any κ value <0.70 to be unacceptable (Vanheule et al., 2014; Spitzer and Fleiss, 1974). These elements further highlight the instability of the diagnostic construct upon which TRD definitions rely.

#### 4.1.2 Symptom-level and biological heterogeneity

Beyond diagnostic categories, qualitative differences in symptom expression appear to map onto partially distinct biological processes. While critiques of categorical diagnosis have been considered excessive in some contexts (Zachar, 2023), growing evidence suggests that specific symptom profiles—particularly neurovegetative symptoms and anhedonia—may be associated with measurable biological differences, including peripheral inflammatory markers (Gasparini et al., 2022). For example, a recent Randomized Clinical Trial (RCT) tested the impact of an inflammatory challenge with lipopolysaccharide (LPS) on anhedonia symptoms in participants with MDD who had high (≥3 mg/L) or low (≤1.5 mg/L) serum C-reactive protein (CRP) concentrations (Savitz et al., 2025). Of interest, depressed individuals with high CRP appeared to be biologically primed to respond more strongly to inflammatory stimuli and with a higher severity of anhedonia (Savitz et al., 2025). This, in turn, could have clinical implications not only for the mere diagnosis but also for response to pharmacological treatment (Gasparini et al., 2022). Antidepressant medications, for instance, may show differential efficacy across symptom domains, with some evidence suggesting greater effectiveness for core mood symptoms compared with sleep or atypical features (Chekroud et al., 2017; Boschloo et al., 2023; Zangen et al., 2023). Taken together, these findings suggest that symptom-level heterogeneity may reflect underlying biological variability with direct implications for treatment response.

#### 4.1.3 Clinical and comorbidity-related heterogeneity

Other sources of heterogeneity, often insufficiently captured by current classification systems, include sex, psychiatric and medical comorbidities, longitudinal course, and psychosocial stressors at onset (Lamers et al., 2011), (Buch and Liston, 2021). Up to 75% of individuals with MDD present with at least one comorbid psychiatric condition (Buch and Liston, 2021), raising questions about shared underlying mechanisms across disorders and the validity of categorical boundaries. These factors contribute to substantial variability in clinical presentation and complicate efforts to define homogeneous patient populations.

#### 4.1.4 Longitudinal heterogeneity

MDD is not only heterogeneous across individuals but also within individuals over time. Longitudinal data, such as those from the Dunedin Study, indicate that individuals may receive multiple different psychiatric diagnoses across the lifespan (Vergunst et al., 2013), challenging the notion of stable categorical disorders. Symptom trajectories are influenced by a complex interplay of genetic, hormonal, and environmental factors, including cumulative exposure to stressors and broader exposome-related influences (Wang et al., 2023).

This temporal instability suggests that vulnerability to MDD and TRD may be better conceptualised as a dynamic, time-dependent process rather than a fixed condition, further complicating attempts to define treatment resistance based on cross-sectional criteria.

#### 4.1.5 Clinical implications

Despite this substantial heterogeneity across diagnostic, biological, and temporal domains, current clinical practice guidelines continue to recommend broadly similar treatment algorithms for MDD, with limited capacity to tailor interventions based on individual symptom profiles or underlying biology. Attempts to identify clinically meaningful subtypes have yielded inconsistent results, with substantial overlap between groups and limited predictive validity for treatment response, aside from a few notable exceptions (e.g., psychotic vs non-psychotic MDD) (Nelson et al., 2018; Arnow et al., 2015).

This mismatch between the complexity of MDD and the relative simplicity of current treatment frameworks represents a fundamental limitation, with direct implications for both clinical management and the validity of TRD as a diagnostic construct.

### 4.2 The limitations of TRD phenotypic definition

The clinical heterogeneity of MDD described above implies that any definition of treatment resistance is already built upon an unstable diagnostic foundation. Building upon this heterogeneous foundation, current TRD introduce additional layers of variability by relying predominantly on the number of failed antidepressant trials. This approach assumes that treatment trials are comparable and adequately conducted, an assumption that is frequently not met in clinical practice (Dizet et al., 2025). These limitations can be broadly conceptualized at three levels: (i) conceptual limitations related to categorical definitions of response and remission; (ii) definitional limitations inherent to the failed trials criterion and its scope; (iii) operational limitations in how these criteria are applied in real-world clinical settings.

#### 4.2.1 Conceptual limitations: Response, remission, and recovery

The classical view on MDD would consider this a fully episodic condition with variably long periods of symptomatic improvement (Marx et al., 2023). However, naturalistic data suggest that this is often not the case (Vergunst et al., 2013). Indeed, a sizeable portion of individuals with MDD experience a waxing and waning of symptom severity and persistence of symptoms well beyond the acute symptomatic phases (Vergunst et al., 2013). The categorical concepts of response, remission, and recovery have been largely borrowed from other fields of medicine, such as oncology, where remission is more easily discerned by the absence of clear signs or symptoms of disease activity, as compared with psychiatry (Rush et al., 2019).

However, despite the limited validity of its definition, there is clear evidence that MDD symptom remission correlates with a better prognostic outcome, and that remitted patients have a lower risk of mood recurrence compared with individuals with only responsive ones, with a higher number of residual symptoms being associated with a higher risk of recurrence (Zajecka MD, 2013). Importantly, the higher the number of failed therapeutic trials, the lower the probability of achieving a remission (Gaynes et al., 2009). However, there is less certainty on the relative weight of the severity and duration of each persisting symptom in influencing the recurrence risk regardless of their number. Arguably, from a purely qualitative perspective, each symptom may also have a different (and potentially greater) impact on functioning and quality of life.

Beyond symptom remission, the overall objective of MDD treatment is recovery, an elusive and difficult target to define, let alone to achieve (Vicent-Gil et al., 2023). In the past, recovery overlapped with sustained clinical remission, defined as the absence of active symptoms of the underlying disorder associated with functional impairment (Vicent-Gil et al., 2023). Albeit inconsistently defined (Rush et al., 2019), the lack of clinical remission after several trials of antidepressant treatments of adequate dose and duration has been labelled TRD.

#### 4.2.2 Definition limitations: the failed-trials criterion and its scope

A central limitation of the current TRD definition lies in the assumption that antidepressant medications constitute a homogeneous pharmacological category (Ghaemi, 2019). This issue is intrinsic to the validity of the failed trials criterion, as it assumes that all treatment trials are equivalent. However, even when considering molecules within the same class (e.g., selective serotonin reuptake inhibitors or tricyclic antidepressants), meaningful differences exist in terms of efficacy and safety profiles (Cipriani et al., 2018), challenging the notion that treatment failures can be directly equated.

The regulatory framework for esketamine illustrates this ambiguity: while allowing the failure of two antidepressant trials of any type, its approved use specifies combination with a selective serotonin reuptake inhibitor (SSRI) or a serotonin-norepinephrine reuptake inhibitor (SNRI), reflecting the context of its clinical development (EPAR, 2019). It also remains unclear whether switching to an alternative medication class after the failure of a first treatment trial leads to better outcomes than switching within the same class, with inconsistent findings across studies (Arnow et al., 2015; Russell et al., 2001; Joyce et al., 2003; Perry, 1996).

Although alternative classification systems based on mechanisms of action or receptor affinity have been proposed as more biologically grounded frameworks (McCutcheon et al., 2023; Caraci et al., 2017), these approaches do not resolve the fundamental issue that treatment trials are not equivalent and therefore cannot be reliably treated as interchangeable units in defining TRD. Taken together, these considerations highlight a core conceptual limitation of the failed-trials criterion, which may oversimplify treatment resistance.

#### 4.2.3 Definitional scope limitations

Beyond pharmacological non-equivalence, the scope of the TRD construct itself presents additional limitations. The apparent loss of therapeutic effect after an initial response, known as antidepressant tachyphylaxis (ATP), has been recognised for several decades (Fornaro et al., 2019; Kinrys et al., 2019) but remains difficult to distinguish from relapse or recurrence, which are often conflated in the literature. On theoretical grounds, ATP assumes an initial response followed by symptom worsening despite ongoing treatment, whereas relapse or recurrence do not require continued pharmacotherapy (Kinrys et al., 2019).

An additional element worth considering is a possible antidepressant-induced clinical worsening of a pre-existing MDD in maintenance treatment (Fava, 2020). Current clinical paradigms encompass treatment-naïve patients, but largely fail to address the possible impact of iatrogenic psychopathological components (Fava, 2020), which may be significant in a subgroup of patients that may ultimately fail to respond to the available pharmacological treatments.

Further compounding this issue, TRD typically refers only to resistance to pharmacological interventions. There is generally no formal requirement to demonstrate a lack of response to neurostimulation techniques (such as electroconvulsive therapy or transcranial magnetic stimulation) or psychotherapy, and definitions tend to focus primarily on acute treatment outcomes. Consequently, such a framework appears ill-equipped to capture individuals who experience a loss of treatment effectiveness over time following an initial response (McAllister-Williams et al., 2020).

The concept of TRD also largely disregards environmental influences on treatment response, often implicitly assuming optimal treatment conditions. However, this assumption may be unrealistic, as evidence suggests that a substantial proportion of individuals with MDD receive suboptimal levels of care (Thornicroft, 2017). Taken together, these considerations have led to the proposal of alternative constructs such as “difficult-to-treat depression” (DTD), defined as depression whose symptoms continue to cause significant impairment despite multiple treatment efforts (McAllister-Williams et al., 2020). While potentially more flexible, this construct remains largely consensus-based and lacks clear data regarding its specificity and objectivity. Therefore, notwithstanding these limitations, TRD is likely to retain a significant role, particularly in regulatory contexts (McAllister-Williams, 2022).

An additional layer of complexity may be offered by more difficult-to-standardise elements pertaining to more subjective clinical predictors of treatment response, including early-life trauma (Williams et al., 2016), suggestibility (Nitzan et al., 2015) and patient preference (McHugh et al., 2013). These factors are rarely incorporated into formal definitions but may substantially influence clinical outcomes.

#### 4.2.4 Operational limitations: assessment, adherence and clinical implementation

Operational limitations further compound these issues, with an overall lack of standardised criteria for key components of TRD definition, including response, remission, treatment adequacy, and adherence. In particular, treatment adherence is rarely formally assessed, despite being a major contributor to pseudo-resistance. Variability in dose optimisation, treatment duration, and the use of augmentation strategies further complicates the interpretation of treatment failure. Several paradigms have been proposed over the years to streamline this process (Sackeim et al., 2019; Gaynes et al., 2020), with the aim of assisting clinicians and researchers alike. We invite the reader to consult an excellent review on the different definitions of TRD for a more in-depth comparative analysis of the different current definitions (McIntyre et al., 2023).

A recent systematic review on the definition of TRD concluded that, despite non-adherence increasingly being recognised as a cause of pseudoresistance, very limited guidance in clinical practice guidelines exists on how to address this problem from a practical perspective (Howes et al., 2022). For instance, none of the available guidance on TRD definition considers applying therapeutic drug monitoring (TDM) for addressing lack of adherence in MDD treatment (Howes et al., 2022; McIntyre et al., 2023)), despite the evidence of relatively good quality supporting its use at least for tricyclic antidepressants (Hiemke et al., 2018) along with some indications that it may be beneficial and sufficiently reliable for some other antidepressants such as sertraline (Hiemke et al., 2018). Given the absence of parenteral formulations for most antidepressants, the lack of structured incorporation of TDM into TRD definitions is noteworthy, particularly considering the potential impact of non-adherence in clinical practice.

In this section, we consider key inconsistencies and limitations of current TRD definitions, with a particular focus on the lack of operationalisation for response and adherence. Table 1 provides a comparative summary aligned with these aims. Current definitions adopted by the European Medicines Agency and the Food and Drug Administration do not provide an operationalised definition of partial response and do not incorporate systematic assessment of pseudo-resistance (McIntyre et al., 2023). Even across alternative definitions, structured operational criteria are rarely specified, with the notable exception of the GSRD–European Group for the Study of Resistant Depression (Bartova et al., 2019; McIntyre et al., 2023), which defines TRD as failure to achieve at least a 50% reduction in total score on the Hamilton Depression Rating Scale (HAM-D) or the Montgomery-Åsberg Depression Rating Scale (MADRS).

**TABLE 1:** Comparative table for different selected TRD definitions with a focus on operationalisation for treatment outcome assessment and medication adherence.

Model assessed	Core criteria	Operationalised criteria to assess response	Operationalised criteria to assess adherence
EMA/FDA Definition (EPAR, 2019; Research C for DE and, 2020)	Inadequate response to ≥2 antidepressants of adequate dose and duration with documented adherence	None specified	No specific assessment for non-adherence and inadequate trial execution
Thase & Rush Staging (Thase and Rush, 1997)	Multi-level staging based on number and type of failed antidepressant trials (Stage I: 1 antidepressant failure; Stage II: Stage I plus failure of an additional antidepressant from another class; Stage III: Stage II plus failure to an adequate trial of tricyclic antidepressant; Stage IV: Stage III plus resistance to an adequate trial of monoamine oxidase inhibitor antidepressant (MAOI); Stage V: Stage IV plus bilateral ECT course failure)	None specified	No specific assessment for non-adherence – reports “adequate trials” of antidepressants
Maudsley Staging Method (MSM) (Fekadu et al., 2018)	Multi-dimensional: (Depressive disorder) number of treatment failures, (Marx et al., 2023) depression severity at baseline, (Author anonymous, 2018) duration of current episode; rates each dimension independently	Severity categories of the Mental and Behavioral Disorders section of the 10th revision of the International Classification of Diseases (ICD-10) – considers in alternative the possible use of validated symptom scales	Accounts for duration and severity, but no operational guidance on how to assess adherence
GSRD Definition (Bartova et al., 2019)	Two consecutive adequate antidepressant trials, characterised by specific clinical predictors (symptom severity, suicidality, psychotic features, anxiety comorbidity)	Operationalise response assessment with validated rating scales	Specify adequacy of trial, but no operational criteria on how to evaluate adherence
Difficult-to-Treat Depression (DTD) (Rush et al., 2022)	Broader concept emphasising chronicity, functional impairment, and complex presentations rather than the sole number of failed trials; includes psychosocial and clinical complexity	The authors discuss multiple outcome domains and emphasise the need to select appropriate measures, but none are specifically endorsed	No standardised or mandatory procedure for systematically verifying adherence.

MBC, defined as the systematic, standardised, and repeated assessment of symptoms using validated rating scales and algorithm-guided treatment approaches provide additional insight into how these limitations may affect clinical practice. Accruing evidence suggests that the routine and systematic measurement of symptom severity using validated scales is associated with more frequent treatment adjustments and improved clinical outcomes compared with unstructured assessment. For example, Guo et al. (2015) reported higher response and remission rates with MBC compared with treatment as usual (TAU), alongside a greater number of treatment adjustments. Similarly, Husain et al. (2025) observed faster and higher remission rates and higher treatment doses in an MBC-based approach. Tables 2, 3 summarise these findings.

**TABLE 2:** Summary of RCTs findings exploring the role of Measurement Based Care in influencing treatment selection and adjustment relevant for TRD definition.

Study and year	Study design	Country	Sample characteristics	Medication changes/Adjustments (MBC/Algorithm vs TAU)
Guo et al. (2015)	RCT, assessor-blinded	China	N = 120 (mean age 47.1 years; 67% female) with MDD diagnosis and HDRS-17 score ≥17	MBC: 44 medication adjustments vs TAU: 23 adjustments (week 2–24); MBC group also had higher antidepressant dosages from week 2–24
Bauer et al. (2009)	RCT, prospective single-centre	Germany	N = 148 inpatients (algorithm-guided standardised stepwise drug treatment regimen- SSTR: n = 74; TAU: n = 74) with ICD-10 major depressive episode with or without psychotic features, dysthymia, longer depressive reaction, and bipolar depression were enrolled.	Algorithm-guided (SSTR): 1.0 ± 1.5 strategy changes vs TAU: 3.0 ± 2.7 changes (p < 0.001) — 3-fold fewer strategy changes with algorithm. SSTR remitters used fewer medications (fixed agents: 1.9 ± 1.1 vs 3.0 ± 1.5; optional: 0.9 ± 0.7 vs 1.5 ± 1.0). Time to remission: 7.0 weeks (SSTR) vs 12.3 weeks (TAU) - log-rank test, χ2MC = 8.64, P = 0.003
Husain et al. (2025)	RCT, assessor-blinded	Pakistan	N = 154 (mean age 34.5 years; 68% female) with nonpsychotic MDD	Survival curves for time to response -log rank test, χ21 = 10.21; P = 0.001 and time to remission -χ21 = 11.08; P = 0.001, with the likelihood of response hazard ratio [HR], 1.53; 95% CI, 1.06–2.20; P = 0.02 and remission HR, 1.80; 95% CI, 1.21–2.68; P = 0.004) along with higher overall treatment doses
Adli et al. (2017)	RCT, 5-arm comparative	Germany	N = 449 inpatients (algorithm-guided arms vs CDES vs TAU)	Algorithm-guided arms (ALGO-DE, ALGO-SW) required fewer total antidepressant medications to achieve remission than TAU/CDES (P < 0.001); shorter time to remission (HR = 1.67 & 1.64). Higher remission rates with ALGO than TAU.
Lin et al. (2012)	RCT, care management program	United States (14 clinics)	N = 214 with depression + poorly controlled diabetes/CHD	In TEAMcare vs usual care, initiation of antidepressant over the year was 3.5 times (95% CI, 2.0–6.3) and 6-fold higher antidepressant adjustment rates in (RR = 6.20; P < 0.001) (either switch or dose adjustments); no difference in medication adherence at 12 months

Abbreviations: AD, Antidepressant; ALGO, standardized stepwise drug treatment algorithms; ALGO-DE, standardized stepwise drug treatment algorithms–dose escalation; ALGO-SW, standardized stepwise drug treatment algorithms -switch medication; CHD, Coronary Heart Disease; HDRS, Hamilton Depression Rating Scale; HR, Hazard Ratio; ICD10 – International Classification of Diseases- 10; MBC, Measurement Based Care; MDD, Major Depressive Disorder; OR, Odds Ratio; PHQ-9, Patient’s Health Questionnaire −9; RCT, Randomized Controlled Trial; RR, Relative Risk; SSTR, standardized stepwise drug treatment regimen; TAU, Treatment As Usual; TRD, Treatment Resistant Depression; VA, Veteran Association

**TABLE 3:** Summary of findings of observational studies relevant for the implications of measurement-based care in the detection of TRD.

Study and year	Study design	Country	Study sample & methods	Key clinical inertia findings
Fife et al. (2018)	Database retrospective cohort	United States (3 health databases)	Administrative claims + pharmacy records; analyzed adequacy of antidepressant dosing	Only 59.6%–66.0% of antidepressant medication eras included ≥1 dispensing at minimum therapeutic dose. 34%–40% of patients never reached adequate dosing before medication was changed/discontinued — implies substantial TRD overestimation due to pseudoresistance
Elbakary et al. (2021)	Retrospective cross-sectional	Qatar	N = 487 MDD patients; 49% (212/431) had initial AD change within ≤30 days; median survival time on initial AD: 43 days	High proportion with very early medication changes suggests inadequate optimization/dose escalation before switching. Younger age, un-optimized dosing, and comorbid anxiety predicted early switching
Liberman et al, (2020)	Retrospective longitudinal cohort	United States (integrated delivery network)	N = 35,246 starting ADs; 7,098 (20.1%) met TRD after mean 402 days (13+ months)	Substantial clinical inertia: mean 13+ months before TRD recognition. Structured patient-reported outcomes (PHQ-9) were documented in only 1.5% of patients; delayed identification of non-response

Abbreviations: AD, Antidepressant; ALGO, standardized stepwise drug treatment algorithms; ALGO-DE, standardized stepwise drug treatment algorithms–dose escalation; ALGO-SW, standardized stepwise drug treatment algorithms -switch medication; CHD, Coronary Heart Disease; HDRS, Hamilton Depression Rating Scale; HR, Hazard Ratio; ICD10 – International Classification of Diseases- 10; MBC, Measurement Based Care; MDD, Major Depressive Disorder; OR, Odds Ratio; PHQ-9, Patient’s Health Questionnaire −9; RCT, Randomized Controlled Trial; RR, Relative Risk; SSTR, standardized stepwise drug treatment regimen; TAU, Treatment As Usual; TRD, Treatment Resistant Depression; VA, Veteran Association

Algorithm-guided approaches further complement MBC by structuring treatment decisions. Bauer et al. (2009) demonstrated faster remission with an algorithm-guided standardised stepwise drug treatment regimen compared with treatment as usual. Overall, these findings suggest that systematic assessment and structured decision-making improve treatment outcomes and reduce variability in clinical management (Zhu et al., 2021; Yang et al., 2021).

Despite this evidence, implementation in real-world practice remains limited. Barriers to the adoption of structured assessment include time constraints, lack of training, and difficulties integrating these tools into clinical workflows (Mayes et al., 2023). This gap contributes to clinical inertia, defined as the persistence of ineffective treatment regimens despite insufficient clinical improvement (Aujoulat et al., 2015; Aujoulat et al., 2014). Declining this issue specifically for the treatment of MDD assumes that maintaining the same treatment regimen for more than 2 weeks might be misguided, as a failure of improvement by at least 20% on routine scale assessments has been shown to be significantly predictive for a lack of further improvements later on (Hicks et al., 2019).

The relatively limited propensity to adopt MBC in clinical practice may somewhat limit the possibility on the part of the clinician to critically address such improvement and apply this knowledge in clinical practice, further prolonging the therapeutic odyssey for users on the one hand and limiting the possibility of identifying TRD on the other, as its definition may critically hinge on the amount of medication prescribed. Poignantly in this regard, Liberman et al., in 2020 described that among 35,246 starting antidepressants from the Electronic Health Record data in Decision Resources Group’s (DRG) Real-World Data, TRD criteria were met in 20% of subjects after an average of 13 months, suggesting a far longer amount of time spent on the same, ineffective treatment regimen, an approach which clearly enough is not coherent with current standard of practice. Interestingly, only 1.5% of individuals included in the analysis had a relevant structured assessment with the PHQ-9, prompting the study authors to hypothesise that the lack of MBC application might have somewhat influenced the level of clinical inertia apparent in this cohort (Liberman et al., 2020).

**FIGURE 1:**
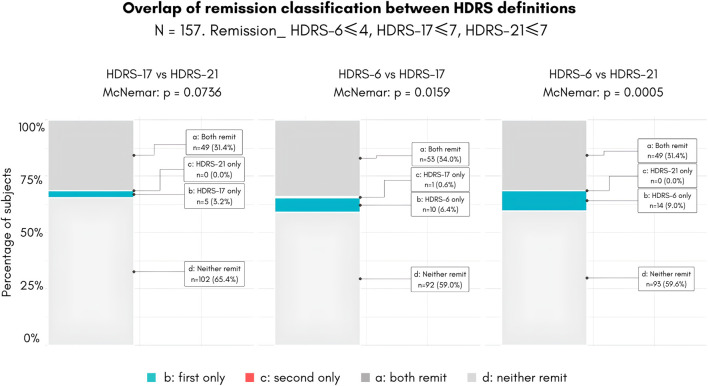
Summary figure on possible pearls and recommendations for identifying and addressing TRD.

Even when structured assessments are employed, important limitations remain. In a sample of 157 patients described in a previous publication from our group, the misclassification between two HDRS subscales (HDRS-6 and HDRS-17) reached approximately 7%, indicating that variability persists even under standardised conditions (Figure 1). It is therefore conceivable that unstructured clinical assessment may introduce even greater heterogeneity in TRD classification. These issues have relevant implications not only for clinical practice but also for translational research, where phenotypic stability is critical.

## 5 Biological and clinical predictors of treatment resistance

### 5.1 The biological underpinnings of TRD

The phenotypic instability of TRD described in Section 4 raises a key question: to what extent can biological evidence help explain, refine, or improve current symptom-based definitions of treatment resistance? In this context, biological findings can be interpreted as (i) illustrating underlying heterogeneity not captured by symptom-based definitions, (ii) moderating treatment response, and (iii) representing potential tools for more precise phenotyping, although their clinical utility remains limited (Otte et al., 2016; Li et al., 2021).

From a genetic perspective, the heritability for severe cases of MDD has been estimated to lie somewhere around 29%–34% (Clements et al., 2021). Although large genome-wide association studies (GWAS) investigating genetic contributors to MDD risk have reported many significant loci, the SNP-based heritability remains relatively modest. One of the largest studies to date on the genetic makeup of TRD, comprising 292,663 participants from the large-scale genomic data from the All of Us (AoU) Research Program (Xu et al., 2025) concluded that 11 of the selected polygenic risk scores (PRS) were found to have stronger associations with TRD, encompassing domains in education, cognition, personality, sleep, and temperament. More specifically, genetic predisposition for insomnia and specific neuroticism traits were associated with increased TRD risk, whereas higher education and intelligence scores were protective. Each susceptibility gene confers a limited increased risk of developing MDD, and ultimately GWAS only allows identification of regions of the genome involved associated with the trait; therefore, it may not necessarily inform understanding of the underlying biology or possible responsible pathophysiological pathways (Ormel et al., 2019). Remarkable attempts to define lifetime risk for MDD using PRSs have yielded promising results (Mitchell et al., 2021). Higher PRS for schizophrenia have been reported in association with poorer response to antidepressants assessed with a self-report instrument single-item question (Nøhr et al., 2022). The association of specific personality traits and PRS have also been investigated in this framework. PRS derived from the Big Five Theory showed that higher PRS for openness and neuroticism were both associated with antidepressant treatment outcome in MDD (Amare et al., 2018). However, despite the ever-increasing population studied, it is increasingly evident that the inherent limitations of this approach may hinder its usefulness in capturing the full breadth of genetic contributions to increased MDD lifetime risk or in aiding clinicians when the diagnosis is unclear (Wray et al., 2021). The same limitations plaguing the TRD definition in clinical practice may partly explain the limitations of population selection for subject selection in translational research (McIntyre et al., 2023). These findings illustrate how genetic evidence may reflect underlying heterogeneity not captured by symptom-based definitions, while also suggesting a potential moderating effect on treatment response. At present, however, their utility as tools for clinical phenotyping remains limited.

A second line of evidence relates to sex-related differences, with female sex appearing disproportionately affected by MDD, however, relatively few studies have specifically focused on trying to disentangle possible underlying factors of this observation, despite women also being at a greater risk for lack of remission and for experiencing residual symptoms (da Silva et al., 2025). These observations further highlight the presence of clinically relevant heterogeneity across biological subgroups, although their role in predicting treatment response or informing phenotyping strategies remains insufficiently characterized.

An additional domain may be traced back to inflammatory patterns and, more specifically, whether peripheral inflammation biomarkers could be harnessed for treatment selection is also unclear at this moment (Strawbridge et al., 2023). Table 4 summarises evidence from RCTs investigating specifically anti-inflammatory treatments in TRD. Overall, studies included in the qualitative analysis do not support a specific, clear benefit for this type of treatment (Hellmann-Regen et al., 2022; Raison et al., 2013; Nettis et al., 2021), with possible preliminary evidence suggesting the need to better stratify subjects for study inclusion based on their inflammatory status (Nettis et al., 2021). These findings are particularly relevant in highlighting the potential of inflammatory markers as tools for more precise phenotyping, while also illustrating how inadequate stratification of biologically distinct subgroups may obscure treatment effects in clinical trials. Taken together, these findings suggest the presence of multiple partially independent biological pathways contributing to TRD, illustrating underlying heterogeneity, moderating treatment response, and highlighting the potential—yet currently limited—utility of biological markers as tools for more precise phenotyping.

**TABLE 4:** Summary of findings of RCTs investigating specifically anti-inflammatory treatments in TRD.

Study, year	Country	Type of study and sample size	Main findings
Hellmann-Regen et al. (2022)	Germany	RCT −168 adult subjects randomised to 200 mg/d minocycline treatment or placebo over a course of 6 weeks with a 6-month follow-up -Inclusion criteria: Adults aged 18–75 years with DSM-5 major depressive disorder, HAMD-17 ≥ 16, CGI-S ≥4, BMI 18–40, inadequate response to at least one standard antidepressant (per MGH-ATRQ), and on stable medication for ≥2 weeks.	No difference with placebo
Nettis et al. (2021)	United Kingdom	RCT- 39 adults with TRD randomised to minocycline or placebo Inclusion criteria: Adults aged 25–60 with DSM-5 non-psychotic MDD (MINI-confirmed), HAM-D-17 ≥ 14 after ≥6 weeks of therapeutic-dose antidepressant (Maudsley-defined) with non-response, CRP ≥1 mg/L, tolerant to current antidepressant and willing to receive minocycline augmentation, stable treatment with no planned changes, and able to provide informed consent. Exclusion criteria: significant active suicidality, bipolar/OCD/eating/PTSD/substance use disorders, warfarin use, recent tetracycline or intolerance, acute infection or autoimmune/inflammatory disease, hepatic or renal failure, unapproved psychotropics, and pregnancy or inadequate contraception.	Some evidence of efficacy if CRP = or > than 3 mg/L.
Raison et al. (2013)	USA	60 subjects consistent antidepressant regimen (n = 37) or medication-free (n = 23) for 4 weeks or more, and who were moderately resistant to treatment as determined by the Massachusetts General Hospital Staging method -randomised to TNF antagonist infliximab (5 mg/kg) or placebo	No difference with placebo

Abbreviations: BMI, Body Mass Index; CGI-S, Clinical Global Impression–Severity; CRP–C, Reactive Protein. HAMD- hamilton depression rating scale; MGH-ATRQ, Massachusetts General Hospital antidepressant treatment response questionnaire; OCD, Obsessive Compulsive Disorder; PTSD, Post-traumatic Stress Disorder; RCT, Randomized Clinical Trial; TNF-alfa–Tumor Necrosis Factor alfa

### 5.2 Clinical, biological and psychosocial predictors of treatment resistance

**FIGURE 2:**
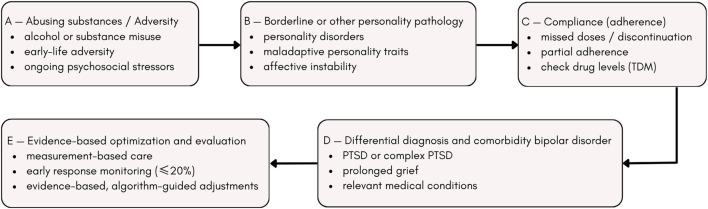
Acronym for the ABCDE mnemonic-heuristic on the steps to follow for adequate and prompt recognition of TRD and how to optimize its management.

While the above findings describe biological correlates of TRD, a related but distinct question concerns which factors predict treatment resistance in clinical settings. Considering the significant burden of disease associated with MDD and the time required to observe symptom improvement even among responders (Gaynes et al., 2009), the early identification of individuals who are likely to respond favourably to pharmacological treatments is of clear clinical relevance. Beyond biological correlates, several clinical frameworks have been proposed to identify predictors of treatment resistance in real-world settings. Among these, the ABCDE heuristic integrates multiple domains (Figure 2).

A—Abusing substances/Adversity. Substance misuse, including alcohol and illicit drug use, may substantially interfere with antidepressant response and adherence. In addition, exposure to early-life adversity and ongoing psychosocial stressors may contribute to symptom persistence and poorer treatment outcomes.

B—Borderline or other personality pathology. Personality disorders, particularly borderline personality pathology, as well as maladaptive personality traits and affective instability, may complicate the clinical presentation and negatively influence treatment response, engagement, and longitudinal course.

C—Compliance (adherence). Suboptimal adherence remains a common and often under-recognized contributor to apparent non-response. This includes missed doses, premature discontinuation, and partial adherence. When clinically appropriate and available, TDM may assist in clarifying adherence and pharmacokinetic issues.

D—Differential diagnosis and comorbidity. Diagnostic reassessment is essential to exclude alternative or comorbid conditions that may mimic or worsen depressive symptoms. Particular attention should be paid to bipolar disorder, post-traumatic stress disorder (including complex PTSD), prolonged grief, and relevant medical conditions.

E—Evidence-based optimization and evaluation. Finally, apparent resistance should not be concluded before ensuring that treatment has been adequately optimised. This includes the routine use of MBC, early response monitoring (e.g., ≤20% symptom reduction), and evidence-based, algorithm-guided treatment adjustments.

#### 5.2.1 Clinical and developmental factors

As previously alluded to in other sections of this review, several additional clinical and biological factors further contribute to variability in treatment response. The complex interplay between early-life trauma and personality disorder may represent a further layer of complexity in influencing treatment response, which may be difficult to account for. For instance, the significant overlap existing between ICD-11-defined complex PTSD disorder and borderline personality disorder is particularly compelling in this regard, as their coexistence may represent a significant cause of TRD in a subset of patients, especially in the most severe cases (Jowett et al., 2020).

Across the lifespan, age-related and neurocognitive factors also appear to influence treatment outcomes. Extreme ages tend to present lower levels of response to monoaminergic agents (Strawn et al., 2023), with possible differences depending on various elements such as possible differences in the underlying neurobiology (i.e., early vs adult-onset MDD), specific age-related stressors, misdiagnosis or indeed diagnostic transition (e.g., prolonged grief disorder (Wen et al., 2022), bipolar disorder (Nestsiarovich et al., 2021)). Somatic and cognitive disorders may be more prevalent in older cohorts, further compounding the heterogeneity of response across the lifespan (Strawn et al., 2023).

#### 5.2.2 Biological and neurocognitive factors

Cognitive dysfunction represents one of the most consistently identified predictors of poor treatment response, with nearly one-third of MDD patients presenting significant cognitive impairment (Hack et al., 2023). Cognitive biotype of MDD may represent a disease subtype, with lower levels of response to standard antidepressants, lower levels of remission and worse psychosocial functioning (Hack et al., 2023). Even in the presence of symptomatic improvement with treatment, MDD with worse pretreatment cognitive performances tends to present persistently greater cognitive impairments (Hack et al., 2023). Especially in the older patient cohort, the misdiagnosis with vascular depression or depression-executive dysfunction may indeed represent a further element of confusion in addressing the evidence for treatment efficacy of standard antidepressants (Aizenstein et al., 2016; Taylor et al., 2018). Interestingly, worse cognitive performances among unaffected relatives of MDD have described. These cognitive signatures may involve a wide range of cognitive performances, from Full-Scale IQ to more specific cognitive domains, and appear to represent an element of a familiar predisposition to develop MDD (MacKenzie et al., 2019). Sex also appears to influence antidepressant response, with the female sex presenting a greater response to antidepressants in general (Strawn et al., 2023; Silva et al., 2023) but possibly also a higher risk of TRD (da Silva et al., 2025), with men presenting a tendency for a better response specifically to tricyclic antidepressants (Silva et al., 2023).

#### 5.2.3 Psychosocial and relational factors

Finally, individual-level psychological and relational factors may further modulate treatment response. Personality traits have also been studied as possible predictors of treatment response. Low harm avoidance, low cooperativeness, and high novelty seeking have all been associated with higher levels of treatment discontinuation (Köhler-Forsberg et al., 2023). Interestingly, other than moderating the efficacy of the antidepressant effect, personality traits such as neuroticism may also be influenced by treatment (Naito et al., 2022; Du et al., 2002). Other elements worth considering may be more intrinsic to the dyadic therapeutic relationship between the service user and the healthcare provider (Wampold and Flückiger, 2023). Its effect has been extensively studied in psychotherapy outcomes (Flückiger et al., 2018); however, it may also play a significant role in other treatment paradigms, and indeed, it has been reported to influence response to antidepressant treatment in MDD (Zilcha-Mano et al., 2015).

However, the predictive value of these factors remains constrained by the instability and inconsistency of TRD phenotyping, as discussed in Section 4. Taken together, these predictors highlight that treatment resistance is not determined by a single domain but rather emerges from the interaction between biological vulnerability, clinical presentation, and psychosocial context, further reinforcing the limitations of purely symptom-based definitions.

## 6 Discussion

The present review highlights that TRD is not a unitary clinical entity, but rather the emergent result of multiple interacting layers of heterogeneity spanning nosological, biological, and operational domains. Section 4 demonstrates that current TRD definitions are largely based on symptom thresholds and treatment counts, both of which are affected by substantial variability in diagnostic practices, treatment adequacy, and adherence assessment. Section 5 further shows that biological findings—including genetic liability, inflammatory markers, cognitive dysfunction, and personality traits—suggest the presence of partially distinct pathophysiological pathways that are only imperfectly captured by current clinical phenotypes.

Importantly, this mismatch has concrete consequences. For example, genetic findings such as the association between polygenic risk scores (PRS) for insomnia or neuroticism and TRD risk, as well as inflammatory studies showing differential symptom expression and treatment response, are difficult to interpret when mapped onto heterogeneous and inconsistently defined clinical populations. Similarly, the modest and inconsistent effects observed in trials targeting specific biological mechanisms (e.g., anti-inflammatory treatments) may partly reflect insufficient phenotypic stratification rather than a true lack of efficacy. Taken together, these observations suggest that phenotypic instability in TRD is not merely a measurement problem but reflects a deeper misalignment between symptom-based definitions and underlying biological heterogeneity.

To address these challenges, we propose conceptualizing TRD within a multi-level framework, in which distinct strategies target different layers of variability.

### 6.1 Level 1: biological stratification

At the most fundamental level, TRD likely reflects biological heterogeneity beneath the symptom surface. Evidence reviewed in Section 5 suggests that multiple domains—including genetic vulnerability (e.g., PRS findings), inflammatory processes (e.g., CRP-associated symptom profiles), and cognitive dysfunction—are associated with differential treatment response. However, these effects are typically modest and inconsistent, limiting their immediate clinical applicability.

A key implication is that biological findings cannot be meaningfully interpreted without more precise phenotypic definitions. For example, inflammatory markers appear to be associated specifically with neurovegetative and anhedonic symptom clusters rather than with global depression severity. Similarly, cognitive dysfunction identifies a subgroup of patients with poorer response to standard antidepressants and worse functional outcomes. These findings suggest that biologically informed stratification should move beyond global diagnostic categories and instead focus on symptom dimensions and clinically relevant subtypes.

Future translational efforts should therefore prioritise: (i) identification of response biomarkers at the level of specific treatments rather than broad drug classes; (ii) integration of cognitive performance, early-life adversity, and personality traits into predictive models; and (iii) development of multimodal approaches combining clinical, biological, and behavioural data. Crucially, progress at this level depends on improving phenotypic definition upstream.

### 6.2 Level 2: improved definition and diagnostic validity of TRD

**FIGURE 3:**
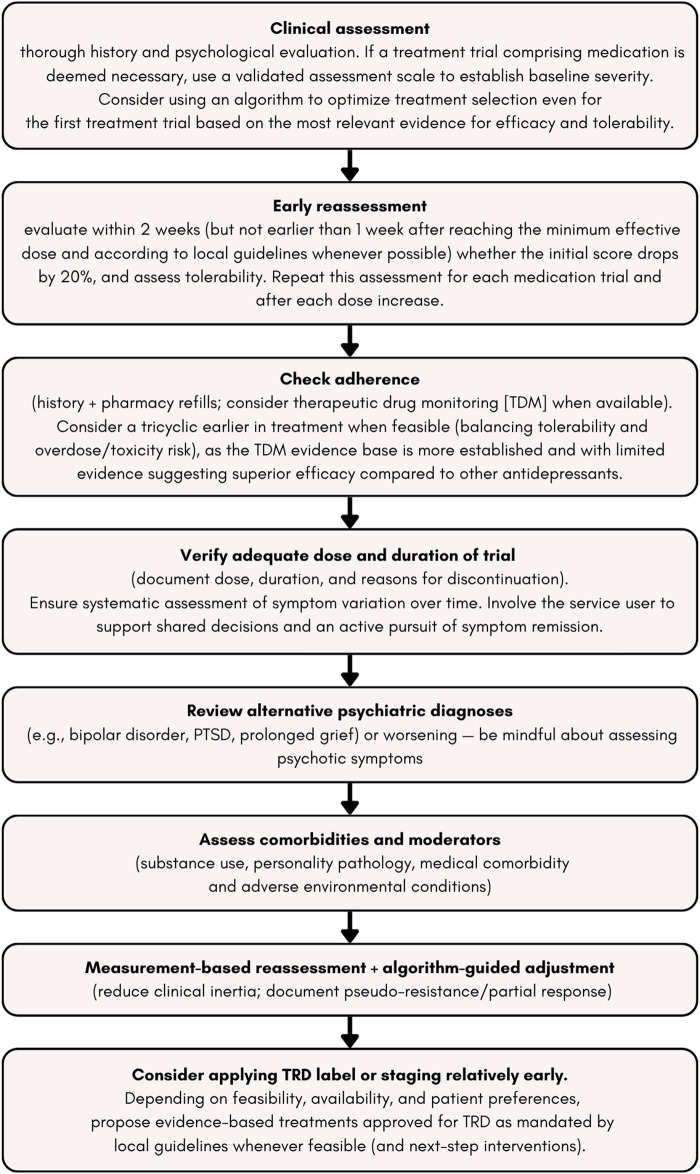
Heterogeneity in remission classification for a convenience sample of 157 iMDD receiving care from an outpatient clinic with differing subscales of HDRS.

Although inconsistencies in the TRD definition are widely acknowledged, current approaches remain largely descriptive and insufficiently operationalized. A central issue is that treatment resistance is defined primarily by the number of failed trials, without systematically accounting for treatment adequacy, adherence, or measurement variability. The evidence reviewed here highlights several concrete challenges. First, treatment adherence is rarely formally assessed, despite being a major contributor to pseudo-resistance. Second, variability in how response and remission are defined introduces additional instability. For example, our analysis showed a 7% misclassification rate between different HDRS subscales even under standardized conditions, suggesting that unstructured clinical assessment may introduce even greater variability. Third, current definitions do not account for phenomena such as tachyphylaxis or iatrogenic worsening, nor do they incorporate non-pharmacological treatment response (Figure 3).

Addressing these limitations requires moving beyond aspirational recommendations toward concrete implementation strategies. These may include: (i) adoption of minimal core outcome definitions across regulatory and research frameworks; (ii) systematic incorporation of adherence assessment, including the use of TDM where feasible; (iii) standardisation of treatment adequacy criteria using structured tools (e.g., ATHF); and (iv) explicit inclusion of multimodal treatment failure (pharmacological and non-pharmacological) in TRD definitions.

However, several barriers must be acknowledged. These include variability across regulatory agencies, limited feasibility in routine clinical settings, lack of clinician training in structured assessment, and the absence of integrated digital systems to support standardization. Overcoming these challenges will likely require coordinated efforts across clinical, regulatory, and research domains, including the development of pragmatic definitions that balance scientific rigour with real-world applicability.

### 6.3 Level 3: measurement-based care

MBC primarily addresses the operational layer of heterogeneity by standardizing how symptoms are assessed over time. Evidence reviewed in Section 4.2 consistently shows that MBC improves clinical outcomes compared with treatment as usual. For example, Guo et al. (2015) reported higher response and remission rates, along with more frequent treatment adjustments, whereas Husain et al., 2025 observed faster and higher remission rates and greater dose optimization with MBC-based approaches. Algorithm-guided interventions further demonstrated accelerated remission compared with standard care.

In addition, early symptom improvement (e.g., ≥20% reduction within the first weeks) has been shown to predict subsequent treatment response, providing a clinically actionable marker for early treatment modification. Despite this evidence, real-world implementation remains limited. Liberman et al. (Liberman et al., 2020) reported that TRD criteria were met only after an average of 13 months, with only 1.5% of patients receiving structured assessment, highlighting substantial clinical inertia.

However, MBC is not a complete solution. Even structured assessments may introduce variability, as illustrated by the observed misclassification between HDRS subscales. Thus, while MBC reduces measurement error and improves clinical decision-making, it does not address underlying biological or nosological heterogeneity. Rather, it provides a more reliable and reproducible framework upon which further stratification strategies can be built.

### 6.4 Level 4: algorithm-guided treatment

Algorithm-guided treatment represents the interface between measurement and clinical decision-making. While MBC improves the quality of symptom assessment, algorithm-based approaches structure how this information is translated into treatment adjustments.

Evidence suggests that algorithm-guided care can accelerate remission and improve treatment adequacy compared with treatment as usual. Importantly, these approaches reduce clinical inertia by promoting timely treatment changes based on predefined criteria. However, like MBC, they remain fundamentally symptom-based and therefore do not address deeper biological heterogeneity.

Their primary value lies in standardising care pathways, improving reproducibility, and ensuring that treatment resistance is identified based on adequately conducted trials rather than prolonged exposure to ineffective interventions.

### 6.5 Integrating clinical and translational perspectives

A key implication of this framework is that clinical and translational priorities are not independent but mutually reinforcing. However, this relationship must be demonstrated rather than assumed.

For example, failure to stratify patients based on inflammatory status may partly explain the inconsistent findings observed in trials of anti-inflammatory treatments. In this context, improved clinical phenotyping—through systematic symptom assessment and identification of biologically relevant subgroups—could enhance the detection of treatment effects. Conversely, biological findings may inform clinical stratification strategies. For instance, the identification of cognitive dysfunction as a predictor of poor response suggests that targeted interventions addressing cognitive impairment may represent a meaningful avenue for personalised treatment.

Similarly, PRS findings indicating associations with neuroticism, insomnia, and cognitive traits highlight the potential for integrating genetic and clinical data into predictive models. However, these approaches remain limited by the instability of the underlying phenotype, reinforcing the need for improved clinical definition as a prerequisite for translational progress.

Taken together, these examples illustrate a bidirectional relationship: improved phenotyping enhances the interpretability and clinical utility of biological findings, while biological insights inform the refinement of clinical classification and treatment strategies. Bridging this gap represents a critical step toward precision psychiatry in MDD.

## 7 Conclusion

Greater efforts should be devoted to improving the homogeneity of the TRD definition to reduce inconsistencies and enhance its ecological validity. The standardisation of TRD phenotyping supported by systematic MBC appears largely endorsed by the available evidence, but is very poorly applied in clinical practice. This element is particularly relevant not only for clinical applications for the evidence in the field but arguably even more in translational research, where the phenotype definition is even more crucial, considering the sizeable underlying heterogeneity to disentangle. Future studies should prioritise harmonised staging approaches and pragmatic implementation strategies capable of bridging the gap between controlled trials and real-world practice. Strengthening the ecological validity of the TRD construct will be critical for advancing precision psychiatry in major depressive disorder.
